# Purification of the Air without Renewing It

**Published:** 1844-02

**Authors:** 


*Purification of the Air without renewing it.*—The *Courier Français* states that a most curious experiment has been lately made in the hospital of the Salpetriére at Paris with a machine invented by Dr. Payerne, called the purifier, the object of which is to purify the air, without renewing it, in hospitals, prisons, mines, diving bells, and, in general, in all places where the air has been vitiated. This experiment, which excited the greatest interest from the important results to be derived from its success, was witnessed by deputies from the Academy of Sciences, the Administration of Hospitals, and by several distinguished chemists and physicians. Dr. Payerne entirely succeeded in what he proposed, viz: to purify the air completely in an enclosed space without communication with the external air. The thermometer, at the same time, descended several degrees. Dr. Payerne proposes, in a few days, to make an experiment with his machine on a diving bell in the Seine, by which the divers may remain an indefinite time under water without communication with the atmospheric air.—*London Med. Gaz.*, Nov. 1843.

